# Immediately scheduled for an appointment to smoking cessation clinics: Key to quitting smoking in chronic airway disease – a multicenter randomized study

**DOI:** 10.18332/tid/204254

**Published:** 2025-06-05

**Authors:** Dilek Karadoğan, Tahsin Gökhan Telatar, İlknur Kaya, Siahmet Atlı, Neslihan Köse Kabil, Feride Marım, Merve Yumrukuz Şenel, Aycan Yüksel, Burcu Yalçın, Ökkeş Gültekin, Merve Erçelik, Metin Akgün

**Affiliations:** 1 Department of Chest Diseases, Recep Tayyip Erdoğan University, School of Medicine, Rize, Türkiye; 2 Department of Public Health, Recep Tayyip Erdoğan University, School of Medicine, Rize, Türkiye; 3 Department of Chest Diseases, Faculty of Medicine, Kütahya Health Sciences University, Kütahya, Türkiye; 4 Department of Chest Diseases, Health Sciences University, Van Education and Research Hospital, Van, Türkiye; 5 Department of Chest Diseases, Yalova Training and Research Hospital, Yalova University, Yalova, Türkiye; 6 Department of Chest Diseases, Faculty of Medicine, Balıkesir University, Balıkesir, Türkiye; 7 Department of Chest Diseases, Faculty of Medicine, Başkent University, Ankara, Türkiye; 8 Department of Chest Diseases, Merzifon Karamustafapasa State Hospital, Amasya, Türkiye; 9 Department of Chest Diseases, Kemalpaşa State Hospital, İzmir, Türkiye; 10 Department of Chest Diseases, Faculty of Medicine, Süleyman Demirel University, Isparta, Türkiye; 11 Department of Chest Diseases, School of Medicine, Ağrı İbrahim Çeçen University, Ağrı, Türkiye

**Keywords:** smoking cessation, lung diseases, obstructive

## Abstract

**INTRODUCTION:**

A significant proportion of patients with chronic airway diseases continue to smoke even after the diagnosis. In addition, smoking cessation support continues to be a neglected issue in real-life settings by physicians for that patient group. Therefore, in our search for a solution to this issue, we conducted our study to evaluate the effect of arranging immediate appointments to smoking cessation outpatient clinics on smoking cessation success in patients with chronic airway disease.

**METHODS:**

This multicenter, randomized, parallel-arm prospective study (NCT05764343) was conducted in pulmonary outpatient clinics between November 2022 and June 2023. Current smoker patients aged ≥18 years diagnosed with COPD, asthma, or bronchiectasis for at least 6 months were included and sequentially randomized in a 1:1 ratio. Both arms received brief smoking cessation interventions, and the intervention arm had immediate access to a smoking cessation clinic appointment. In contrast, the control arm received a standard quitline appointment for routine service. The primary endpoint was the self-reported smoking cessation rate at 3 months, analyzed using an intentionto-treat approach.

**RESULTS:**

The study comprised 198 patients in the immediate appointment arm and 199 in the usual care arm. The quit rate was significantly higher in the immediate appointment arm (26.7%) than in the usual care arm (16.5%, p=0.014). Access to smoking cessation medication was 69.3% in the intervention group against 22.0% in the control group (p<0.001). Multivariable analysis identified access to smoking cessation medication as the sole significant predictor of cessation success at 3 months (adjusted odds ratio, AOR=5.64; 95% CI: 2.89–11.03).

**CONCLUSIONS:**

Our study revealed that access to evidence-based smoking cessation support is positively associated with successful quitting. Compared to the usual care arm, the immediately appointment-scheduled arm has a higher access rate of cessation support. Therefore, smoking cessation support, including pharmacotherapy, should be part of routine care for patients with chronic airway diseases.

**CLINICAL TRIAL REGISTRATION:**

The study is registered on the official website of ClinicalTrials.gov Identifier: ID NCT05764343

## INTRODUCTION

Tobacco exposure is a major contributing factor to numerous diseases that cause premature death. Among these, chronic airway diseases are particularly burdensome. Chronic obstructive pulmonary disease (COPD) is the third leading cause of death worldwide. Tobacco exposure also plays a critical role in the progression of asthma, the most prevalent chronic airway disease, where the benefits of prevention are well-established. Bronchiectasis similarly suffers devastating impacts from tobacco use^1^. Despite these risks, a significant proportion of patients with this condition continue to smoke: about 40% of those with COPD and 11–20% of asthma patients^1-4^. In one cohort, only 15.8% of bronchiectasis patients had never smoked, and there was a significant association between smoking, all-cause mortality, and lung cancer-related mortality risks^5^. Further evidence suggests smoking is a factor in the onset of bronchiectasis in young adults^6^.

Research on smoking cessation in COPD and asthma patients typically focuses on the efficacy of various treatments^7^. Studies have mostly compared the effectiveness of varenicline, nicotine replacement therapy (NRT), and bupropion against a placebo. Regarding non-pharmacological approaches, intensified counseling has been found to be more effective than usual care^7^. Smoking cessation interventions during acute COPD exacerbations in hospitalized patients have also shown effectiveness^8^. However, it has been reported that physicians are not sufficiently engaged in turning these teachable moments into an advantage for smoking cessation assistance, especially in this group of patients^9^.

The 5As method (Ask, Advise, Assess, Assist, Arrange) is considered the gold standard for brief tobacco cessation interventions, with Ask-Advise-Refer (AAR) and Ask-Advise-Connect (AAC) being feasible alternatives in routine care. The AAC approach has been found more effective than AAR, yet information on these methods’ impact on smokers with COPD or asthma remains limited^10,11^. Our previous research shows that smoking cessation support remains a neglected issue in real-life settings for both asthma and COPD patients^1^. Health systems should continue to seek solutions for sustainable tobacco cessation programs by implementing policies to facilitate routine cessation interventions, removing cost barriers, and integrating cessation aid into routine care^12^. Ottawa Model for Smoking Cessation (OMSC) is an example of a successful system-level intervention and informed many international and modified national projects for cessation support of hospitalized patients^13-15^.

Immediate cessation interventions in lung health control programs have been shown to be more effective compared to usual care^16,17^. Studies evaluating the effect of immediate or proactive support on cessation success in patients with chronic airway disease are limited in number^18,19^. We aimed to evaluate the impact of providing immediate cessation support through instant appointment at a cessation clinic on quit success in patients with chronic airway diseases.

## METHODS

This study was a multicenter, randomized, parallelarm, prospective trial (NCT05764343) conducted from November 2022 to June 2023. Ethical approval was granted by the Institutional Review Board of Recep Tayyip Erdoğan University Clinical Research Ethics Committee. Patients were recruited from chest disease outpatient clinics. Data collection commenced in November 2022, and the final patient recruitment was completed in March 2023.

The detailed study protocol is described in a previous article where we present the preliminary analyses^18^.

### Inclusion and exclusion criteria

Patients aged ≥18 years, diagnosed with asthma or COPD or bronchiectasis for at least 6 months, current smokers (defined as having smoked at least 100 cigarettes in their lifetime and continuing to smoke daily or on some days), accessible via phone for follow-up calls 3 months post-randomization and patients who provided written informed consent, were included in the study. The presence of active psychiatric disorders, impaired cognitive functions, and currently receiving smoking cessation treatments were the exclusion criteria.

### Randomization and allocation

Participants were sequentially randomized in a 1:1 ratio to either the control or intervention arm upon identification and after providing written informed consent. The control group received a brief smoking cessation intervention and was advised to secure appointments at smoking cessation outpatient clinics via quitlines, reflecting standard practice. Conversely, the intervention group received the same brief intervention but was also immediately scheduled for an appointment at the smoking cessation outpatient clinic.

Eligible patients implemented brief tobacco cessation interventions. The intervention group (immediate support arm) received smoking cessation interventions. Patients were immediately scheduled for an appointment at the smoking cessation outpatient clinic. The appointments of the patients were organized to be within one week. The rate of attendance to the scheduled appointment was also followed up after 1 week. Patients in the usual care arm also received brief smoking cessation interventions. Afterward, they were advised to apply to smoking cessation outpatient clinics by making an appointment through the Quitline or the Ministry of Health appointment systems, which is a routine practice.

### Follow-up and monitoring

One week after the intervention, participants in both arms were contacted via phone by their physicians to assess their smoking cessation efforts and admissions to smoking cessation clinics. The outcomes of the first week were published as preliminary results^18^.

A follow-up call was made at 3 months to all participants to evaluate their quit status, cessation clinic attendance, and the use and duration of any pharmacological smoking cessation treatments. Counseling and free pharmacotherapy access are available at smoking cessation clinics^12^.

### Outcome measures

*Access to smoking cessation medication*

Participants were considered to have accessed evidence-based smoking cessation treatments if they initiated pharmacotherapy approved for smoking cessation, such as nicotine replacement therapy (NRT) or bupropion, at the follow-up at 3 months.

*Quitter*

Successful quitting was defined as sustained abstinence from smoking since the target quit date. Quitting status was determined by self-report and confirmed at the follow-up at 3 months. Smoking cessation was confirmed by self-report.

### Statistical analysis

Data analysis was conducted using IBM SPSS Statistics for Windows (Armonk, NY, USA, IBM Corp.). Numerical data are presented as means and standard deviations, while categorical data are shown as frequencies and percentages. The chi-squared test was utilized to examine relationships between categorical variables. The normality of continuous data was assessed using the Kolmogorov-Smirnov and Shapiro-Wilk tests. Differences between groups for non-normally distributed data were analyzed using the Mann-Whitney U test.

A multivariable model was built based on biological plausibility. A logistic regression model was developed to identify factors influencing the 3-month smoking cessation rate, adjusted for education level, Fagerström test score, FEV_1_%, and unscheduled visits. The model was first run with missing values, and no imputation was used. A second model was developed for the analysis of the intention to treat. A two-tailed p<0.05 was considered statistically significant in all analyses.

For the intention-to-treat analysis, the missing value mechanism is considered Missing Completely at Random (MCAR). Missing values were only present in the endpoint variable of smoking cessation success. There were 13 missing values in this variable. Nine of these were in the usual care arm, and four were in the immediate appointment arm. All missing values are between the ages of 40 and 69 years. Five of the missing values are female and eight are male. The distribution of missing values by disease type was 8 COPD, 5 asthma, and 0 bronchiectasis. Missing values were filled using multiple imputation methods. As a result of missing value analysis, the Markov Chain Monte Carlo method with a maximum of 10 iterations was used as a multiple imputation method. SPSS software was used with five imputations and a maximum of 10 iterations. All analyses were performed with a multiple imputation dataset. SPSS 29 was used for data analysis. SPSS performs pooled analyses on multiple imputed datasets using Rubin’s Rule.

## RESULTS

### Randomization and comparison between arms

**Figure 1:**
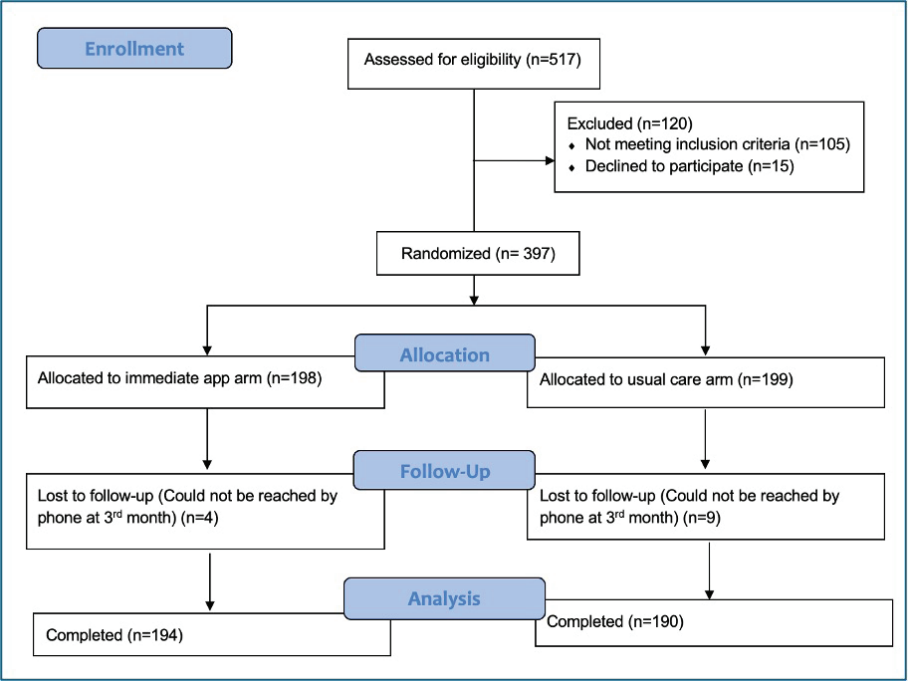
Flow diagram of recruitment. Multicenter, prospective, randomized, open-label study, conducted between November 2022 and June 2023 at pulmonary outpatient clinics in Türkiye (N=397)

The analysis included data from 397 patients: 198 in the immediate appointment arm and 199 in the usual care arm (Figure 1). The mean age was 52.7 ± 13.1 years in the immediate appointment group and 54.4 ± 13.1 years in the usual care group. In terms of sex, the immediate support group consisted of 58 females (29.3%) and 140 males (70.7%), while the usual care group had 72 females (36.2%) and 127 males (63.8%). Patient characteristics across the two arms are detailed in Supplementary file Table 1^18^, showing no significant differences except in education level, Fagerström test for nicotine dependence (FTND) scores, FEV_1_ % predicted, and unscheduled doctor visit numbers.

### Comparisons of outcome measures between study arms

**Figure 2:**
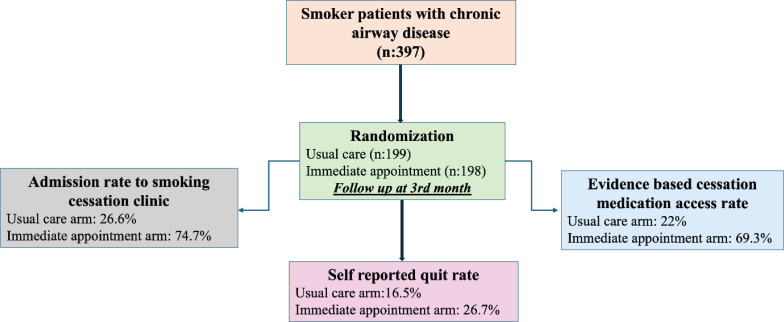
Main outcomes of the study. Multicenter, prospective, randomized, open-label study, conducted between November 2022 and June 2023 at pulmonary outpatient clinics in Türkiye (N=397)

Figure 2 and Supplementary file Table 2 present data on access to smoking cessation support and quit rates at 3 months post-randomization. The quit rate was significantly higher in the immediate appointment arm at 26.7%, compared to 16.5% in the usual care arm (p=0.014). Furthermore, 69.3% of the immediate support group accessed evidence-based smoking cessation medications, significantly more than the 22.0% in the usual care arm (p<0.001); mean duration of smoking cessation medication use was also longer in the immediate appointment arm than in the usual care arm (p<0.001).

### Associated factors with quit status in the third month

Table 1 examines the factors associated with a successful quit attempt in the third month. After adjusting for confounders, access to evidence-based smoking cessation pharmacotherapy was positively associated with quit success (AOR=5.64; 95% CI: 2.89–11.03).

**Table 1:** Multivariable logistic regression models for associated factors with successful quit at third month. Multicenter, prospective, randomized, open-label study, conducted between November 2022 and June 2023 at pulmonary outpatient clinics in Türkiye (N=397)

	*Total n*	*Quitter (N=86) n (%)*	*Non-quitter (N=311) n (%)*	*AOR*	*95% CI*	*p*
**Age** (per unit age increase)				1.01	0.97–1.03	0.661
**Sex**						
Male ®	267	61 (22.8)	206 (77.1)	1		
Female	130	24.6 (18.9)	105.4 (81.0)	0.57	0.28–1.17	0.130
**Airway disease**						
COPD ®	220	43 (19.5)	177 (80.5)	1		
Asthma	165	36.6 (22.1)	128.4 (77.9)	1.11	0.51–2.38	0.785
Bronchiectasis	12	6 (50.0)	6 (50.0)	2.04	0.49–8.43	0.324
**Onset age to smoking** (per unit age increase)				1.03	0.98–1.07	0.212
**BMI** (per unit increase)				0.96	0.90–1.07	0.232
**Randomization arm**						
Immediate appointment arm ®	198	52.8 (26.6)	145.2 (73.3)	1		
Usual care arm	199	32.8 (16.4)	166.2 (83.5)	0.81	0.43–1.53	0.529
**Access to smoking cessation medication**						
Not-accessed ®	215.8	22.2 (10.3)	193.6 (89.7)	1		
Accessed	181.2	63.4 (34.9)	117.8 (65.0)	5.64	2.89–11.03	**<0.001**

AOR: adjusted odds ratio. The model was adjusted with the following variables: education level, Fagerström test score, FEV_1_%, and unscheduled doctor visits. ® Reference categories. The frequency values in the table belong to pooled values obtained as a result of intention-to-treat analysis.

## DISCUSSION

Our study demonstrates that access to smoking cessation support significantly enhances quit success in individuals with chronic airway diseases. The provision of immediately scheduled appointments to cessation clinics markedly improved cessation rates, highlighting the pivotal role of accessibility to evidence-based interventions. Furthermore, our findings underline the impact of certain factors on the likelihood of quitting, including the critical importance of access to support. These insights advocate for the integration of immediate, evidencebased smoking cessation strategies within patient care protocols.

Based on real-life data, it has been found that the rate of access to evidence-based smoking cessation therapies for those with chronic airway disease is extremely low^1^. It is therefore important to examine other solutions to make it easier for these patients to access this help. The COPD guideline emphasizes that smoking cessation assistance, and even a short 3-minute cessation intervention, is highly effective in helping COPD patients to quit smoking^20^. Although the role and influence of physicians on this issue are very important, it has been reported that physicians lack participation in providing smoking cessation support to COPD patients^9^. The availability and use of cessation interventions among smokers with COPD have improved in recent years, but there are still some gaps that contribute to a lack of physician engagement with tobacco cessation support. Policy interventions can effectively increase the accessibility and utilization of smoking cessation treatments. Appropriate policies can help make cessation intervention more routinely provided in health systems and remove cost and access barriers for patients^7^. Smoking cessation outpatient clinic services have become widespread, and exemplary practice has been established in Türkiye. Access to free smoking cessation pharmacological treatments is also provided through these outpatient clinics^12^. Referral of patients to smoking cessation outpatient clinics by their primary physicians is an effective method for quit success. On the other hand, accessibility difficulties and the need to improve the service standards of that clinic have been reported^1,21^. Effective tobacco cessation support requires a comprehensive approach provided by all health disciplines^22^. However, there are limited data on the provision of this support. It is known that courses on tobacco cessation in medical school curricula are insufficient worldwide^23^. Tobacco cessation support is mostly provided by pulmonologists^24^. However, very few pulmonologists are trained or certified to provide tobacco cessation clinic services, so the majority do not provide cessation clinic support^25^. Our study points to the effectiveness of providing smoking cessation assistance by pulmonologists, who are responsible for the management of the group of patients who experience the greatest burden of smoking. Therefore, in light of our findings, we argue that all pulmonologists should provide smoking cessation assistance to their patients and that it should become part of the routine.

The primary endpoint, the smoking cessation rate, was 26% in the immediate appointment arm and 16% in the usual care arm. In this study, what we call immediate support is actually similar to the previously tried AAC intervention. The usual care arm is similar to the AAA intervention^10,[Bibr CIT0011]11^. The rate of access to evidence-based cessation help increased as we provided the ‘Arrange’ stage more effectively by giving immediate appointments. In order to scale this up, it is important that physicians who can provide smoking cessation help and these services become widespread and part of the routine. Effective use of the brief cessation intervention is also key. We did not initiate immediate smoking cessation pharmacological treatments; we only effectively implemented the brief intervention. There are also examples of immediate initiation of tobacco cessation treatments^16,17^. In a novel cessation approach, samples were recruited from among high-risk individuals for lung cancer presenting for lung health screening and randomized into two arms regardless of their motivation to quit smoking. At follow-up at 3 months, the immediate intervention group had a higher rate of smoking cessation (21.1% vs 8.9%)^[Bibr CIT0017]17^. Another study by the same researchers was a single-blind, randomized controlled trial of smokers participating in a lung health screening. On randomly assigned days, smokers received immediate counseling support and pharmacotherapy by a health professional trained in smoking cessation, while the other group received routine services (brief smoking cessation advice and an appointment at a smoking cessation center). As a result, quit rates at 3 months were 29.2% versus 11%^16^. Among chronic airway diseases, for smoker COPD patients, quitting smoking is reported to be more challenging than for smokers without COPD due to greater nicotine dependence, lower self-efficacy, and lower self-esteem. The presence of depression is more common in smokers with COPD. Despite these adverse conditions, if effective time and resources are dedicated to smoking cessation, long-term quit rates of 14% to 27% have been reported^20^.

### Strengths and limitations

The strengths of our study include its multicenter, randomized design and the implementation by pulmonologists in real-life settings, addressing a previously neglected issue within smoking cessation research. Also, it shows that actively engaging people and setting up appointments may result in better engagement in smoking cessation services and treatment than those who passively are offered care. There is also significant improvement even after adjusting for known confounders, some of which were unbalanced, such as education level. However, a strong limitation of our study is the reliance on self-reported data for smoking cessation, without validation through carbon monoxide (CO) measurement, which may affect the accuracy of the reported quit rates. Another limitation is that some variables, such as Fagerström test score, education level, and FEV_1_%, were not homogeneously distributed among the randomized groups. In order to minimize their effect on the primary outcome, a multivariable logistic regression model was applied to adjust for these variables.

## CONCLUSIONS

Our study shows that access to evidence-based smoking cessation support significantly (5.64-fold) increased cessation rates in patients with chronic airway disease. The rate of access to evidencebased smoking cessation support was higher in the immediate appointment scheduled group than in the usual care group (69.3% vs 22.0%). Therefore, a timely approach is needed for effective tobacco cessation support. Smoking cessation support should be part of routine care for patients with chronic airway disease and physicians should be competent to provide pharmacological cessation treatments.

## Supplementary Material



## Data Availability

The data supporting this research are available from the authors on reasonable request.
