# Supplementary material for: Immediately scheduled for an appointment to smoking cessation clinics: Key to quitting smoking in chronic airway disease – a multicenter randomized study
Source: Tob Induc Dis. 2025 Jun 5;23:10.18332/tid/204254. doi: 10.18332/tid/204254 (PMC12139391; doi:10.18332/tid/204254)
Supplement: Supplementary file 1 [file TID-23-76-s1.pdf]

Supplementary file Table 1. Baseline characteristics of study population and comparisons according to randomization arm[1]. Multicenter, prospective, randomized, open-label study, conducted between November 2022 and June 2023 at pulmonary outpatient clinics in Türkiye (N=397)

	**Usual care (n:199)**	**Immediate appointment arm (n:198)**	**p**
**Age,** mean (SD)	54.4 (13.1)	52.7 (13.1)	0.251
**Sex**			0.144
**Female**	72 (36.2)	58 (29.3)	
**Male**	127 (63.8)	140 (70.7)	
**BMI (Kg/m^2),** mean (SD)	27.0 (5.03)	26.7 (4.45)	0.738
**Education level**			0.003
**Primary schooling**	99 (49.7)	78 (39.4)	
**Secondary schooling**	34 (17.1)	29 (14.6)	
**High school graduate**	48 (24.1)	47 (23.7)	
**University graduate**	18 (9.0)	44 (22.2)	
**Income level**			0.185
**≤ Minimum wage**	122 (61.3)	124 (62.6)	
**Up to 3 folds of minimum wage**	62 (31.2)	50 (25.3)	
**> 3 folds of minimum wage**	15 (7.5)	24 (12.1)	
**Diagnosis duration,** mean (SD)	6.20 (7.10)	6.21 (7.11)	0.709
**Smoking pack/year,** mean (SD)	36.8 (24.3)	38.7 (26.7)	0.739
**Smoking duration in years, mean (SD)**	33.6 (15.1)	32.0 (14.8)	0.383
**Previous assisted quit attempts, mean (SD)**	0.6 (1.8)	0.3 (1.1)	0.139
**Previous non-assisted quit attempts, mean (SD)**	1.3 (2.7)	1.1 (1.7)	0.981
**Fagerström score,** mean (SD)	5.78 (2.74)	6.50 (2.85)	0.011
**Presence of depression diagnosis**	36 (18.1)	32 (16.2)	0.610
**Presence of anxiety diagnosis**	31 (15.6)	44 (22.2)	0.091
**Presence of other comorbidities**	104 (52.3)	87 (43.9)	0.097
**FEV_1/FVC,** mean (SD)	72.4 (12.8)	73.7 (13.6)	0.166
**FEV_1 %,** mean (SD)	66.2 (21.5)	72.0 (22.6)	0.039
**Airway disease**			0.266
**COPD**	106 (57.6)	114 (57.6)	
**Asthma**	89 (44.7)	76 (38.4)	
**Bronchiectasis**	4 (2.0)	8 (4.0)	
**GOLD Categories of COPD patients**			0.980
**A**	31 (29.2)	34 (29.8)	
**B**	43 (40.6)	47 (41.2)	
**E**	32 (30.2)	33 (28.9)	
**Asthma severity**			0.060
**Mild**	39 (44.3)	44 (57.9)	
**Moderate**	42 (47.7)	31 (40.8)	
**Severe**	7 (8.0)	1 (1.3)	
**Unscheduled doctor visits in last year,** mean (SD)	0.74 (1.29)	1.02 (1.36)	0.003

Abbrevations: COPD: Chronic Obstructive Pulmonary Disease, BMI: Body mass index, FEV_1: Forced expiratory volume in first second, FVC: Forced vital capacity.
The usual care group received a brief smoking cessation intervention and was advised to secure appointments at smoking cessation outpatient clinics via quitlines. Immediate appointment group received brief intervention and immediately scheduled for an appointment at the smoking cessation outpatient clinic.

Supplementary file Table 2. Comparisons according to smoking cessation interventions of both arms. Multicenter, prospective, randomized, open-label study conducted between November 2022 and June 2023 at pulmonary outpatient clinics in Türkiye (N=397)

	**Usual care (n:199)**	**Immediate appointment arm (n:198)**	***p***
**Quit status at 3rd month.**			**0.014**
**Quitter**	32.8 (16.5%)	52.8 (26.7%)	
**Non-quitter**	166.2 (83.5%)	145.2 (73.3%)	
**Admission to smoking cessation clinic at 3rd month**			**<0.001**
**Admitted at least once**	53 (26.6%)	148 (74.7%)	
**Did not admitted**	137 (68.4%)	46 (23.2%)	
**Evidence-based smoking cessation medication**			**<0.001**
**Accessed**	43.8 (22.0%)	137.4 (69.3%)	
**Not accessed**	155.2 (77.9%)	60.6 (30.6%)	
**Used medication**			**<0.001**
**Nicotine replacement therapy**	18 (9.0%)	81 (40.9%)	
**Bupropion**	23 (11.5%)	54 (27.2%)	
**Not used any**	149 (74.8%)	59 (29.7%)	
**Duration of smoking cessation medication use, mean (SD)**	14.0 (18.4)	22.1 (16.6)	<0.001

1. Karadoğan D, Telatar TG, Kaya İ, et al. Effectiveness of immediate appointment scheduling in smoking cessation clinics for patients with chronic airway diseases: Preliminary results from a randomized trial. Tob Induc Dis. 2024;22:10.18332/tid/191782. doi:10.18332/tid/191782

© 2025 Karadoğan D. et al.
